# The roles of genes in the bitter taste

**DOI:** 10.3934/genet.2019.4.88

**Published:** 2019-12-24

**Authors:** Harem Othman Smail

**Affiliations:** Department of Biology, Faculty of science and health, Koya University Koya KOY45, Kurdistan Region-F.R. Iraq

**Keywords:** TAS2R38, bitter taste receptors, phenylthiocarbamide (PTC), chromosomes 7, TAS2R, homozygotes, heterozygotes, polymorphisms

## Abstract

The aims of this review were to understand the roles of bitter taste genes in humans. Some of the peoples have the capacity to taste some chemical substance such as phenylthiocarbamide (PTC) while others cant not based on the dietary hazards and food preferences. There are two alleles responsible to express these phenotypes which are homozygous recessive. In human TAS2R38 genes located on the chromosome number 7 and consist of different nucleotide polymorphism that related to detection of the phenotype of different chemical compounds such as 6-n-propylthiouracil (PROP) and phenylthiocarbamide bitterness and this Gene is the member of the TAS2R genes which are eleven pseudogenes and twenty that has roles in many biological processes.

There are many factors that affect the bitter taste such as food, age, sex, and different diseases. The mechanism of food bitter taste and genotype of TAS2R38 until know not well understood due to that the proof of relation between bitter taste sensitivity and food is harmful. there are many different diseases can impact the influence of taste such as neoplasm and lifestyle such as consumption of alcohol along with the use of medication, head trauma, upper tract infections. On the other hand, A relation between TAS2R38 genotype and meal preferences has been observed among children, however, no associations have been mentioned among older adults. Some previous research proved some vital points that show an association between type 1 of diabetes and phenylthiocarbamide (PTC) but other studies cannot demonstrate that. However, of other disease such as obesity is controversial but other studies reported to the relationship between them.

## 1. Introduction

People vary in their capacity to perceive their environment, and person variations in vision and hearing are mechanically assessed and, when needed, humans are given help to compensate for their deficiencies, i.e., they are supplied eyeglasses or listening to aids. Compared with these two senses, individual differences in taste are given much less attention and are now not assessed without in instances the place humans take part in a lookup find out about or have gone to their doctor with unique complaints about taste loss [1]. Taste perception is a key element in the interplay between organisms and the environment, as it presents essential data about the quality and nutritional value of the meals ate up and approves human beings to observe viable dietary hazards. Humans can perceive five major different tastes: Sweet, bitter, sour, salty and umami (the taste of L-glutamate) [2].

Genetically mediated sensitivity to bitter taste has been related to food preferences and consuming conduct in adults and children [3]. Alleles in the taste 2 receptor member TAS2R38 have been linked to the potential to discover bitterness in bitter-tasting compounds and in many foods, and humans with these bitterness sensitivity alleles have been proven to be less in all likelihood to eat alcohol, presumably due to the fact of alcohol's bitter taste [4]. Bitter taste receptors (Tas2Rs) are a subfamily of G-protein coupled receptors expressed not only in the oral cavity however additionally in various extra-oral tissues and disorder states. Several natural bitter compounds from plants, such as bitter melon extract and noscapine, have displayed anti-cancer results against quite a number of cancer types [5].

Bitter taste receptors (taste family 2 bitter receptor proteins; T2Rs), found in many tissues outside the tongue, have currently come to be practicable therapeutic objectives [6]. Bitterness is a taste sensation that arises when precise chemical substances come into contact with receptors in specialized cells on the human tongue. But not anybody perceives the equal bitterness for a given stimulus; this character variant is partly genetically decided and can have an effect on meal perception, preferences, and consumption [7]. The bitter taste receptors (TAS2Rs) activated via these polyphenols and therefore the half-maximum effective concentrations (EC50) of each agonist–TAS2Rs combine had been determined [8].

## 2. History of bitter taste

More than 70 years in the past A.L. Fox stated that phenylthiocarbamide (PTC) tastes extraordinarily bitter to some humans (defined as “tasters”) however not bitter at all to others (“non-tasters”). Since then, several families, twins, and population research have shown that the incapability to taste PTC is inherited in an almost Mendelian recessive manner [9]. Variation in the potential to taste the artificial compound phenylthiocarbamide (PTC) used to be first identified in the early 1930s, when A. J. Fox observed the polymorphism in himself and a coworker, organic chemist C. R. Noller [10].

The capability to taste phenylthiocarbamide (PTC) is a basic phenotype that has long been acknowledged to range in human populations. This phenotype is of genetic, epidemiologic, and evolutionary hobby due to the fact the capability to taste PTC is correlated with the capability to taste other bitter substances, many of which are poisonous [11]. It used to be mentioned over sixty-five years in the past that chimpanzees, like humans, differ in taste sensitivity to the bitter compound phenylthiocarbamide (PTC). This was once advised to be the end result of a shared balanced polymorphism, defining the first, and now classic, example of the results of balancing choice in great apes [12].

## 3. Genes related to the bitter taste

At the molecular level, bitter taste is mediated by means of taste 2 receptors (Tas2rs). Studies on Tas2r have made the main advances in the latest years [13]. Human TAS2R genes are positioned on chromosomes 7 are rather polymorphic. Among the recognized genetic variants, the diplotype of TAS2R38, consisting of three single-nucleotide polymorphisms (SNPs) [A49P (145G > C, rs713598), V262A (785T > C, rs1726866) and I296V (886A > G, rs10246939)], has been well described for its purposeful modifications (Choi and Kim, 2019). The TAS2R s harboring the perfect ranges of genetic variety appear in an area unique the TAS2R30-31 cluster, which consists of TAS2R30, -31, -43, -45 and -46. Genes in this cluster comprise several SNPs related to taste phenotypes such as sensitivity to the bitterness of synthetic sweeteners and phytotoxins [14]. Polymorphisms in the TAS2R38 gene supply perception to phenotypes long related 6-n-propylthiouracil (PROP) and phenylthiocarbamide bitterness [15].

## 4. Genotype of bitter taste

Differences in genotypes fluctuate with recognize to amino acid substitutions encoded at certain positions on some of the bitter receptor proteins. So far, the most studied human taste receptor in the TAS2R bitter taste receptor family is TAS2R38 (Sandell and Collado 2018). In most of these studies, PTC taste sensitivity used to be described as a bimodal autosomal trait inherited in an easy Mendelian recessive pattern [16]. Receptors for human bitter taste are encoded with the help of the TAS2R gene family that contains twenty-five useful genes and eleven pseudogenes that are subject to biological process forces. The most studied gene in this family is TAS2R38, which encodes a receptor that mediates the capability to taste the bitter compounds phenylthiocarbamide (PTC) and 6-n-propylthiouracil (PROP) [17].

The PROP taste phenotype varies notably amongst individuals, influencing eating behavior and therefore may also play a function in body composition. This variation is related to polymorphisms in the bitter receptor gene TAS2R38 and the taste-bud trophic factor Gustin gene [18]. Despite the latest development in the useful expression of hT2Rs in vitro, up till now, hT2R38, a receptor for phenylthiocarbamide (PTC), used to be the only gene immediately linked to variants in human bitter taste [19]. Phenylthiocarbamide (PTC) and 6-n-propylthiouracil (PROP), chemically related compounds, are probes for genetic variant in bitter taste, even though PROP is safer with much less sulfurous odor [20]. The potential to taste phenylthiocarbamide (PTC) indicates complicated inheritance in humans [21].

Research has proven that molecular diversity in the TAS2Rs of human beings and different primates leads to functional variations in individuals' bitter taste perception. The exposure to the particular flora of a geographic place is thinking to be essential using pressure of choice on TAS2Rs. Humans pick out bitterness when precise chemical compounds contact unique receptors on the apical surface of taste cell membranes. There are about 25 bitter receptors in the human genome, and some of their ligands have been recognized via cell-based assay and transgenic techniques Genetic affiliation research have additionally provided clues about receptor-ligand pairs Currently, not all bitter chemical substances have a regarded receptor and not all receptors have a known ligand [22].

## 5. Bitter taste and food

Eating is a critical part of an individual's well-being and daily-life practices. The greater palatable a meal is, the extra probably it will be eaten. Thus, meals first-rate perceived with our senses is a vital factor contributing to our diet and health [23]. The presence of some healthful phytochemicals in meals can be paired with high bitterness, and buyers have a tremendous avoidance of bitter-tasting food. This reasons a hole between preferences and wholesome needs of customers [24]. Genetic variations in bitter taste perception may additionally account for man or woman variations in meal preferences. Other factors such as age, sex and ethnicity can also regulate the response to bitter-tasting compounds. There are several participants of the TAS2R receptor gene family that encode taste receptors on the tongue, and genetic polymorphisms of TAS2R38 have been related to marked differences in the understanding of PTC and PROP. However, the affiliation between TAS2R38 genotypes and aversion to bitter-tasting meals is now not clear [25].

## 6. Mechanism of bitter taste Gene

Bitter taste grasp evolved as a key detection mechanism towards the ingestion of bioactive substances and is mediated using TAS2R gene family individuals invertebrates. The most notably recognized and amazing studied bitter substance is phenylthiocarbamide (PTC), which is recognized through TAS2R38 and has a molecular structure related to that of glucosinolates contained in Brassica plants. The “non-taster” phenotypic polymorphism (i.e., now not touchy to PTC-containing foods) has been recognized in many primates, such as people [26]. The genes and the food selections are intently connected. Taste and olfaction receptors may have advanced collectively at some stage in vertebrate evolution. The high frequency of genetic variations inside taste and olfactory receptors are special in the human genome Therefore, variations in these two perceptions can also be viewed as a landmark of human evolution [27]. The sensory properties of foods information meals preferences and intake, importantly deciding dietary and fitness status. Variations in sensitivity to the bitter taste of phenylthiocarbamide (PTC) and different thioamide-containing compounds are properly documented. However, there is combined proof involving the association between bitter taste sensitivity, food preferences and metabolic danger factors [28].

## 7. Bitter taste and poisons in vegetation

Some investigators hypothesize that this sense gives information so that humans do no longer ingest bitter-tasting poisonous chemical compounds. Potent poisons are discovered in some vegetation (e.g., like ricin and castor beans) which render them inedible [29]. However, for many different plants, the efficiency or quantity of toxin is low enough so that even even though some (e.g., turnips or cabbage) would possibly taste bitter, they can be eaten with fewer consequences Taste has affected one's preference of foods. It permits one to select the food one likes most. Some diseases such as liver diseases, neoplasm, and lifestyle such as consumption of alcohol along with the use of medication, head trauma, upper tract infections and exposure to toxicant substances have been reportable to considerably influence taste [30].

Chronic cigarette smoking can also affect chemosensory function, which in turn, might also affect cigarette usage. Because menthol in cigarettes can attenuate nicotine bitterness, choice of menthol/nonmenthol cigarettes may additionally be influenced by using the capability to become aware of bitterness [31]. Polymorphisms in bitter taste receptor gene TAS2R38 alter the potential to feel the depth of bitterness of phenylthiocarbamide (PTC) and 6-n-propylthiouracil (PROP). Genetic variants in sensitivity closer to PTC and PROP may also affect food preferences and susceptibility to certain diseases [32]. A relation between TAS2R38 genotype and meal preferences have been observed among children, however, no associations have been mentioned among older adults. This, alongside with the decreased capability to taste certain ingredients with growing age, suggests that environmental factors are extra necessary than genetic influences in food preferences among the elderly [33].

## 8. Detection of bitter trait

Nontasters (tt) comprehend their genotypes because they have to be homozygous recessive to express their phenotype. Such is not the case for tasters (T-), and the tasters in the type will be curious about whether they are homozygotes (TT) or heterozygotes (Tt). Until lately there have been two strategies to answering this question: one that includes the usage of the classification allele frequencies to calculate the conditional chance that a taster in the type is of homozygous and heterozygous, and some other that provides pedigree evaluation to the determination of genotype changes [34].

Taste discernment lets in the evaluation of the chemical elements in food to decide whether or not they comprise nutrients or toxins. Currently, six fundamental tastes have been recognized and broadly widespread along with salty, sweet, sour, bitter, fat, and umami [35],[36]. The capacity to taste phenylthiocarbamide (PTC) and the structurally associated compound 6-e-propylthiouracil (PROP) is one of the excellent investigated human phenotypes. These compounds are typical due to the fact of their capability to generate a bitter taste in some persons however not in others [37]. About 75% of human beings can taste phenylthiocarbamide (PTC), whilst the other 25% can't taste it as it has been demonstrated in many countries all through the world [38].

A 0.13 g of PTC used to be weighed with a sensitive digital balance, and then used to be dissolved in one hundred ml of distilled water to prepare solution No.1, which represents the perfect concentration, and attended a sequence of gradual dilution [39]. Individuals who detected a bitter sensation following placement of a 3.80cm × 1.43cm strip of filter paper impregnated with 0.007mg of PTC (Carolina Biological Supply Company) for a minimal of 5 seconds had been regarded PTC “tasters” for the reason of this experiment. Previous research has proven that this test is dependable [40] even though it does not distinguish between subclasses of tasters, such as medium PTC tasters and PTC supertasters, as described through responses along sectors of a bitter intensity rating scale [41].

## 9. Bitter taste and disease

Recent research has now proven that taste receptors are additionally expressed a long way beyond the tongue, from the airway and gastrointestinal epithelia to the pancreas and brain. The features of many of these so-called extraoral taste receptors continue to be unknown, however rising fundamental science and medical proof suggests that bitter and sweet taste receptors in the airway are essential in sensing bacteria and regulating innate immunity [42]. The lack of ability with taste PTC has been related **to** a wide variety of clinical illnesses not normally related to taste impairment.

## 10. Bitter taste in Parkinson disease

Abnormalities in the function/expression of G protein-signaling pathways have been implicated in PTC perception and additionally in dopamine expression and regulation in Parkinson's disease. No, find out about has but probed whether PTC tasting is disrupted in Parkinson's disease (PD) [43]. The substance 6-n-propylthiouracil (PROP) tastes bitter to some humans however is tasteless to others. An individual's PROP taster repute is physiologically determined. In the present body of literature, there is some disagreement regarding sex variations in PROP taster status. Some research has pronounced that women are greater sensitive to PROP while others did not discover this impact. Several research have proven that PROP sensitivity, as a measure of taste responsiveness, is linked to food acceptance, eating behavior, food preferences, and even personality traits [44].

## 11. Bitter taste in other disease

A wide variety of preceding research had been carried out on the relationship between diabetes and PTC taste perception. Some of these researches reported tremendous interactions between lack of ability to taste PTC and DM others mentioned the lack of an association between PTC taste blindness and DM [30]. T2R38 is a bitter taste receptor expressed in the sinonasal tract, and nonfunctional alleles of this receptor have been implicated in treatment-refractory Cystic fibrosis in non-Cystic fibrosis patients [45]. Previous research exhibit that young people who are sensitive to the bitter taste of 6-n-propylthiouracil (PROP) record extra frequent consumption of sweets and much less popular intake of meats (savory fats) relative to teenagers who are PROP insensitive. Laboratory research is wanted to verify these findings [46]. The relationship between the PROP, PTC taste phenotypes, and obesity is controversial, and some however not all reviews recommend that nontasters have extended BMIs. Early reports from Fisher et al., stated that PTC tasters tended to be thinner (ectomorphs), whereas nontasters tended to have heavier body kinds [47].

Bitter agonists selective for taste receptor type 2 (TAS2Rs), TAS2R5 and TAS2R10 influenced ghrelin secretion in fundic cells. The stimulatory impact of the widely tuned bitter agonist, denatonium benzoate, was once selectively blunted using obesity in the small gut however not in the fundus [48]. The type 2 family of taste receptors (T2Rs) reply to bitter tastants. These receptors are expressed for the duration of the gastrointestinal (GI) tract, with place dependant roles. In the oral cavity, T2Rs are concerned in the aware perception of bitter tastants, while in the decrease GI tract they have roles in chemoreception and regulation of GI function. Through these various roles, these receptors may also be concerned in modulating the urge for food and diet, with consequences for weight regulation and obesity [49]. The bitter taste receptor T2R38 has currently been confirmed to adjust upper airway innate protection and can also affect affected person responses to therapy [45].

Detection and identification of bitter compounds draw excellent interest in the pharmaceutical and food industry. Several standard agonists of precise bitter taste receptors have been determined to showcase anti-cancer effects. For example, N-C=S-containing compounds, such as allyl-isothiocyanates, have proven cancer chemo-preventive effects. It is worth noting that the human T2R38 receptor is particular for compounds containing N-C=S moiety [50]. Taste and odor changes, generally stated in cancer and different acute and persistent illnesses [51] In a latest study, taste and odor modifications had been existing in nearly half of treatment-naïve patients with solid tumors Most additionally had different dietary symptoms and had been frequently at dietary threat [52].

## 12. Conclusions

From this review I reached the following conclusions: (1). Ability of peoples to bitter taste are different from one to others; (2). The genes and receptor has vital roles in the bitter taste; (3). In some diseases may be changes bitter taste receptor and expressed.
